# Cross-Sectional Associations of Screen Media Use Patterns with Executive Functioning and Emotional Self-Regulation Among U.S. Preschool-Aged Children

**DOI:** 10.21203/rs.3.rs-11221493/v1

**Published:** 2026-10-04

**Authors:** Soyang Kwon, Bridget Armstrong, Sarah B Welch, Courtney A. Zulauf-McCurdy

**Affiliations:** Northwestern University; University of South Carolina; Northwestern University; Ann & Robert H. Lurie Children’s Hospital of Chicago

## Abstract

**Background:**

Children’s screen media use increases substantially during the preschool years, a period of rapid development in executive functioning and emotional self-regulation. Greater screen time has been associated with difficulties in both domains, but most studies have focused on total screen time. This approach may obscure distinct associations with developmental domains because screen media use varies in activity (video viewing vs. digital game playing) and device type (mobile devices vs. television). We examined whether associations between screen media use and executive functioning and emotional self-regulation differed by activity and device type among preschool-aged children.

**Methods:**

A study design was cross-sectional. The study sample consisted of 238 mother-child dyads with children aged 3–4 years participating in the Behavioral and Emotional Development in Early Childhood Study (BEES). Mothers reported child media use using the Media Assessment Questionnaire, which assessed daily minutes spent viewing video content and playing digital games on mobile devices and television, along with background television and reasons for allowing screen use. Primary outcomes were executive-function difficulty measured using the Childhood Executive Functioning Inventory and emotional self-regulation difficulty using the Emotion Regulation Checklist. Multivariable linear regression models estimated associations between screen media use and the outcomes, adjusted for child age, child sex, maternal education, and neighborhood opportunity.

**Results:**

Children averaged 109 minutes/day (SD = 92) of screen time, including 60 minutes/day (SD = 40) of television video viewing, 30 minutes/day (SD = 39) of mobile video viewing, 13 minutes/day (SD = 25) of mobile game playing, and 6 minutes/day (SD = 20) of television game playing. Total video viewing was correlated with background television (ρ = 0.48; p < 0.001), and mobile video viewing was correlated with parental use of screens to calm the child (ρ = 0.50; p < 0.001). Each additional 30 minutes/day of total screen time was associated with greater executive-function difficulty (B = 0.03; 95% CI = 0.01, 0.06), but not emotional-regulation difficulty. In decomposition analyses, longer video viewing was associated with greater executive-function difficulty (B = 0.06; 95% CI = 0.01, 0.11), whereas longer mobile-device use was associated with greater emotional-regulation difficulty (B = 0.05; 95% CI = 0.01, 0.08).

**Conclusions:**

In this cross-sectional examination of U.S. preschool-aged children, greater video viewing was associated with greater executive-function difficulty, whereas greater mobile-device use was associated with greater emotional-regulation difficulty. Findings highlight the importance of considering activity and device type when examining the impact of screen media use on child development.

## Introduction

Digital media are now embedded in the daily lives of young children in the United States (U.S.), as mobile devices such as smartphones and tablets have expanded access to media content.^1–3^ A 2024 national survey reported that children aged 2–4 years used screen media for an average of 2 hours and 8 minutes per day.^3,4^ High levels of screen media use in young children are a significant public health concern, because greater screen time has been associated with poorer cognitive and behavioral outcomes in this age group.^5,6^ Preschool is a particularly important period for understanding the developmental implications of screen media use because executive functioning and emotional self-regulation develop rapidly during these years^7,8^ and are important for later learning, social functioning, and school readiness.^9–13^

Children’s screen media use is heterogeneous with respect to both the activities children engage in (watching video content vs. playing digital games) and the devices they use (televisions vs. mobile devices). Screen media use also occurs in different contexts (e.g., co-using with a caregiver) and may serve different purposes (e.g., keeping children occupied, calming them when they are upset). Yet much of the literature characterizes children’s screen media exposure primarily by total duration, collapsing potentially distinct forms of use into a single measure.^5,14,15^ This approach may obscure meaningful differences in how screen use relates to child development because the relevance of screen use may depend on its content, context, technology type, and mode of engagement.^15^ Examining these dimensions separately may reveal associations that are obscured by total screen time and help clarify which aspects of screen media use are most relevant to child development.

Evidence linking screen media use to executive functioning in young children is mixed, and part of this inconsistency may reflect differences in how screen media use is measured and categorized. A meta-analysis of 15 studies including children under 6 years found no association between total screen time and executive functioning.^16^ In another meta-analysis of screen use contexts, program viewing was negatively associated with cognitive outcomes (r = − 0.16) whereas game or app use was not.^5^ These findings suggest that examining screen media use by activity type may provide greater insight than total screen time alone.

Screen media use has also been linked to psychosocial development, commonly through externalizing and internalizing behavior and social-emotional competence outcomes.^5,6^ Emotional self-regulation, the ability to manage emotional arousal and respond adaptively to frustration and distress,^17^ may be particularly relevant to mobile device use, as they are portable and available on demand, allowing them to fill brief intervals that would otherwise require a child to wait, self-entertain, or tolerate mild frustration, the everyday occasions through which regulatory skills are practiced.^18,19^ Radesky et al.^18^ found that more frequent use of mobile devices to calm an upset child was associated with greater emotional reactivity among children aged 3–5 years. However, this finding reflects a specific context of mobile device use, and evidence on associations between device-specific screen media use and emotional self-regulation remains limited.

The aim of this study was to examine associations between screen media use and executive functioning and emotional self-regulation among US children aged 3 to 4 years. We first assessed associations between total daily screen media use and developmental outcomes. We then evaluated whether these associations differed by media activity (video viewing vs. digital game playing) and device type (mobile devices vs. television). Finally, we characterized family media context by examining correlations between types of screen media use and reported contexts and reasons for children’s screen media use.

## Methods

### Study design and setting

We established the Behavioral and Emotional Development in Early Childhood Study (BEES), a longitudinal cohort study of mother-child dyads in the Chicago metropolitan area, designed to investigate how young children’s physical behaviors (i.e., physical activity, screen media use, and sleep) are related to cognitive and social-emotional development. The present analysis used baseline data.

### Participant eligibility and recruitment

BEES enrolled children aged 3 or 4 years and their mothers. Children were eligible if they lived in Illinois and had no cerebral palsy or other medical condition that would preclude physical activity. Participating mothers were required to live with the child at least 50% of the time and be able to communicate in English.

Participants were recruited between October 2025 and September 2026 through two pathways. First, families who had previously participated in the Child and Mother Physical Activity Study (CAMPAS)^20^ were invited by email to continue their participation in BEES. CAMPAS was a cohort study conducted in the Chicago metropolitan area from August 2022 to June 2026 to examine physical activity behaviors from ages 12–36 months. Second, additional families with eligible 3- to 4-year-old children were recruited through targeted outreach in Illinois ZIP codes classified as having low or very low metropolitan Child Opportunity Index (COI) levels, an indicator of neighborhood resources and opportunities for children.^21^ Electronic recruitment flyers were distributed to local libraries, pediatric clinics, and childcare centers within these target ZIP-code areas. We also identified potentially eligible children aged 3–4 years residing in the target ZIP codes through a local health system database and sent email invitations. This targeted recruitment strategy was intended to increase socioeconomic diversity in the BEES sample. The BEES protocol also permitted enrolled families to refer potentially eligible friends or family members.

Families interested in participation completed a REDCap-based eligibility screener. Study staff contacted potentially eligible mothers by email to schedule a telephone call and offered either in-person or remote participation. All participants selected remote participation. During the call, staff completed the informed consent process, including maternal consent for the mother’s participation and parental permission for the child’s participation, administered a brief participant profile questionnaire, and provided instructions for the online maternal survey and child accelerometer wear. The present analysis included participants who completed the baseline maternal survey.

### Data collection

Mothers received a secure survey link by email and completed the survey in REDCap. The full survey battery required approximately 30 minutes and assessed demographic and family characteristics, parenting practices, and child screen media use, sleep, executive functioning, and emotional and behavioral functioning. Demographic information included child age, sex, race and ethnicity, childcare attendance, maternal educational attainment, and residential address. Residential addresses were used for geocoding to determine census tract-level COI.

### Exposure: Screen media use

We used the mother-reported Media Assessment Questionnaire (MAQ)^2^ to assess child screen media use. The MAQ characterizes young children’s media exposure across activity type, device type, and context. Specifically, it assesses daily minutes spent on mobile viewing, television viewing, mobile gaming, and television gaming.^2^ In this study, we used modified response options for each item, ranging from 0 to ≥ 150 minutes/day in 15- or 30-minute increments, with the highest category coded as 150 minutes. This upper category was selected to capture the range of typical preschool screen use (approximately 2 standard deviation [SD] above the mean reported in prior studies^22^) while limiting respondent burden from an excessive number of response options. The ≥ 150-minute category was coded as 150 minutes, its lower bound. To capture the family context of media use, we also used MAQ items on caregiver-child co-use, household background television, and parents’ reasons for allowing screen use (i.e., to educate my child; to calm my child down; to keep my child busy; my child enjoys; to spend time together), each rated on a 5-point Likert scale (0 = never to 4 = very often).

For the present analysis, we examined screen media use at multiple levels: (1) total screen time, (2) video viewing versus digital game playing (activity type), (3) mobile versus television use (device type), and (4) the four device-by-activity combinations of mobile viewing, television viewing, mobile gaming, and television gaming. For each device-by-activity item, daily minutes were calculated as [(weekday minutes × 5) + (weekend-day minutes × 2)]/7. Daily screen viewing was then calculated as the sum of mobile and television viewing, and daily screen gaming as the sum of mobile and television gaming. Mobile-device use was calculated as the sum of mobile viewing and mobile gaming, and television use as the sum of television viewing and television gaming. Total screen time was calculated as the sum of all four device-by-activity variables. Because each device-by-activity item was capped at 150 minutes per day, activity- and device-level variables were capped at 300 minutes, and total screen time at 600 minutes per day.

### Outcomes: Executive functioning and emotional self-regulation

The two primary outcomes were executive functioning and emotional self-regulation. Executive functioning was assessed using the mother-reported Childhood Executive Functioning Inventory (CHEXI).^23,24^ CHEXI is a caregiver-report measure of everyday executive-function diddiculties comprising working-memory and inhibition domains, with 24 items rated on 5-point Likert scale (1 = definitely not true to 5 = definitely true). It has demonstrated good reliability and a two-factor structure corresponding to working memory (13 items) and inhibition (11 items),^24^ and this structure and measurement invariance have been replicated in large diverse samples of preschool-aged children across countries.^23,25^ We calculated working-memory and inhibition scores as the mean of items in each domain, following prior scoring approaches.^26^ Given the study’s focus on overall executive functioning, we calculated a composite CHEXI executive-function diddiculty score as the mean of the two domain scores.^27^ Higher CHEXI scores indicated greater executive-function diddiculties.

Emotional self-regulation was assessed using the mother-reported Emotion Regulation Checklist (ERC), a 24-item caregiver-report measure with two subscales.^28,29^ The 8-item Emotion Regulation subscale assesses adaptive regulation, including situationally appropriate affective displays, empathy, and emotional self-awareness, while the 15-item Lability/Negativity subscale assesses lack of flexibility, mood lability, and dysregulated negative affect.^28^ Mothers rated each item from 1 (rarely/never) to 4 (almost always). Both subscale scores were calculated by averaging their respective items after appropriate reverse scoring according to published ERC procedures.^28^ For analytic consistency, the Emotion Regulation subscale was then reverse-scored to create an Emotion Dysregulation score, such that higher scores on both scales indicated greater diddiculties. A composite ERC emotional-regulation diddiculty score was calculated by averaging the 23 items after appropriate reverse scoring,^30^ so that higher composite scores indicated greater emotional-regulation diddiculties.

We also examined broader social-emotional functioning as a secondary outcome using the mother-reported 25-item Strengths and Diddiculties Questionnaire (SDQ).^31,32^ Items were rated on a 3-point scale (0 = not true, 1 = somewhat true, and 2 = certainly/very true), with positively worded items reverse-scored. Following the scoring manual,^31^ the externalizing score was created by summing Conduct Problems and Hyperactivity/Inattention subscales and the internalizing score by summing Emotional Symptoms and Peer Problems subscales. The total diddiculty score was calculated as the sum of the Emotional Symptoms, Conduct Problems, Hyperactivity/Inattention, and Peer Problems subscales, with higher scores representing greater social/emotional diddiculties.

### Statistical analysis

All analyses were conducted using R version 4.4 (R Foundation for Statistical Computing, Vienna, Austria). Participant characteristics and screen media use were summarized using frequencies and percentages for categorical variables and means and SD for continuous variables. Distributions of outcome variables were examined using histograms and normal quantile–quantile plots. Internal consistency of each multi-item measure was evaluated using Cronbach’s alpha. Spearman correlation coeddicients between screen media use measures and family media-context variables were calculated.

Before fitting parametric models, we examined the shape of each screen exposure–outcome association using locally estimated scatterplot smoothing (LOESS), a nonparametric method that does not assume a specific functional form.^33^ In a guideline-based analysis, children were classified as having low (≤ 60 minutes/day) or high (> 60 minutes/day) total screen time based on the recommended limit for this age group.^19^ The developmental outcomes were compared between the two groups using two-sample t-tests.

Multivariable linear regression models examined associations between screen media use and continuous developmental outcomes. Screen use variables were scaled so that coeddicients represented differences associated with an additional 30 minutes/day of the corresponding screen exposure. This 30-minute increment was selected based on prior screen-time intervention studies that reported reductions of approximately 30 minutes/day.^34–38^ Outcomes were modeled on both original and standardized (mean of 0 and SD of 1) scales. The original-scale coeddicients were reported to provide interpretation in the units of each instrument, whereas standardized-scale coeddicients were reported to facilitate comparison of the magnitude of associations across outcomes measured on different scales. Models were adjusted for child age in years, child sex (male vs. female [reference group]), maternal educational attainment (< 4 vs. ≥4-year college degree [reference group]), and census tract-level COI category (very low or low, moderate, vs. very high or high [reference group]). Statistical significance for the primary outcome analyses was evaluated using a Bonferroni-adjusted two-sided α of 0.025. Regression coeddicients were presented with 95% confidence intervals (CIs).

Regression assumptions were evaluated using residual and influence diagnostics, including residual-versus-predicted plots, normal quantile–quantile plots, studentized residuals, leverage statistics, and Cook’s distance. Diagnostics indicated approximately linear relationships, no substantial heteroscedasticity, and residual distributions consistent with normality apart from modest tail deviations.

### Power consideration

A priori power calculations were based on the two primary developmental outcomes.

Power was evaluated for detecting a single coeddicient in a multivariable regression model containing six predictors. The target effect size was a partial R^2^ of 0.05 (partial *r* = 0.22 and Cohen’s *f^2^*=0.053), corresponding to screen media exposure explaining 5% of the outcome variance after adjustment for covariates. Assuming SD of 60 minutes/day for screen media exposure,^39^ this effect size corresponds approximately to a 0.11-SD difference in the developmental outcome per additional 30 minutes/day of screen use. A total sample of 184 participants was estimated to provide 80% power at Bonferroni-adjusted two-sided significance level of 0.025.

## Results

A total of 263 families consented, of whom 238 (90.5%) contributed the survey data used in the current report. Children were predominantly 3 years old (77.7%) and evenly distributed by sex (49.6% male; Table 1). Children averaged 109 (SD 92) minutes/day of total screen time. Video viewing on television accounted for most exposure (60 [40] minutes/day). One hundred thirty-five children (56.7%) reportedly did not play digital games at all. Owing to an implementation delay, ERC administration began on February 11, 2026, resulting in a smaller analytic sample for ERC-derived outcomes (n = 186). Internal consistency for the CHEXI Working Memory and Inhibition subscale was high (Cronbach’s α = 0.89 and 0.82, respectively). Internal consistency for the ERC scales was also high (Cronbach’s α = 0.85).

### Family media context and patterns of screen media use.

Total screen time was most strongly correlated with the frequency of background television (Spearman ρ = 0.50), followed by use of screens to calm the child (ρ = 0.44; Fig. 1). Video viewing was most strongly correlated with background television (Spearman correlation ρ = 0.48, p < 0.001). Mobile video viewing was most strongly correlated with use of screens to calm the child down (ρ = 0.50, p < 0.001).

### Bivariate analyses between screen media use and developmental outcomes.

Of 238 children, 148 (62.2%) exceeded 60 minutes/day of total screen time. Developmental outcomes did not differ significantly between high/low screen time groups for any outcomes (Table 2). LOESS plots (Fig. 2) showed generally increasing patterns across much of the observed exposure range, with nonlinear patterns and widening uncertainty at higher exposure levels.

### Adjusted associations between screen media use and developmental outcomes.

As LOESS plots suggested possible curvature, both linear and quadratic models of screen time were tested. Because the quadratic term was not statistically significant for any outcome (all p ≥ 0.13), a linear model was used as the final model. As shown in Table 3, each additional 30 minutes/day of total screen time was associated with a 0.03-point higher executive-function diddiculty score (95% CI = 0.01, 0.06; Table 3). The association with the emotional-regulation diddiculty score was not statistically significant.

When associations were examined by activity type (Table 4), video viewing was associated with higher executive function diddiculty (B = 0.06; 95% CI = 0.01, 0.11; standardized β = 0.09 SD; 95% CI = 0.02, 0.17; Supplementary Table 1), whereas digital game playing was not (B = − 0.01; 95% CI = − 0.08, 0.06). Neither digital game playing (B = 0.05; 95% CI = 0.00, 0.10) nor video viewing (B = 0.00; 95% CI = − 0.03, 0.03) was associated with emotional-regulation diddiculty scores. When associations were examined by device type (Table 5), mobile-device use was associated with higher emotional-regulation diddiculty scores (B = 0.05; 95% CI = 0.01, 0.08) and higher social/emotional diddiculty score (B = 0.47; 95% CI = 0.07, 0.86), corresponding to 0.13 SD (95% CI = 0.04, 0.22) for emotional-regulation diddiculty scores and 0.09 SD (95% CI = 0.01, 0.17) for social/emotional diddiculty scores per 30 minutes/day of mobile-media use (Supplementary Table 1).

Statistical significance was evaluated using a Bonferroni-adjusted threshold of *p* < 0.025; significant associations are shown in bold.

CHEXI, Childhood Executive Functioning Inventory; CI, confidence interval; COI, Child Opportunity Index; ERC, Emotion Regulation Checklist; SD, standard deviation; SDQ, Strengths and Diddiculties Questionnaire.

Analyses for CHEXI subscales demonstrated that video viewing was significantly associated with worse working memory but not inhibition (Supplementary Table 2). Digital game playing and mobile-device use were significantly associated with higher ERC Lability/Negativity scores, but not with ERC Emotion Dysregulation scores (Supplementary Table 3). For SDQ subscales, digital game playing was significantly positively associated with internalizing scores, and mobile-device use was significantly positively associated with externalizing scores (Supplementary Table 4).

## Discussion

In this cross-sectional study of 238 U.S. preschool-aged children, longer total screen time was associated with greater parent-reported executive-function diddiculty, but not with emotional self-regulation. Decomposing screen media use revealed that these associations did not arise uniformly across activities (i.e., video viewing vs. digital game playing) or devices (i.e., mobile device vs. television). Higher video viewing, but not digital game playing, was associated with greater executive-function diddiculty. In contrast, higher mobile-device use was associated with poorer emotional self-regulation. Family media context also varied by type of screen use with video viewing most strongly associated with background television, while mobile-device use was most strongly associated with parents using screens to calm the child.

### Executive functioning.

Longer total screen time was associated with greater parent-reported executive-function diddiculty (standardized β = 0.05 SD per 30 minutes/day; partial r = 0.15). This association was driven primarily by video viewing (β = 0.09 SD; r = 0.16) and was not observed for digital game playing or device types. The activity-specific findings are consistent with a meta-analysis^4^ showing that program viewing was associated with poorer cognitive outcomes (r = 0.16), whereas digital game playing was not, although that review combined executive functioning with language and academic outcomes. Our findings are also consistent with a recent cross-sectional study^33^ of 359 Canadian children aged 3–4 years in which show/movie/video viewing was associated with poorer executive functioning. However, that study found stronger associations with response inhibition than working memory (r = 0.20 vs. r = 0.14), whereas the association in our study was more evident for working memory. The Canadian study also reported that digital game playing was associated with better working memory and that greater mobile-device use was associated with poorer inhibition and working memory, patterns that were not observed in the present study.^33^ Taken together, these findings provide relatively consistent evidence that video viewing, rather than digital game playing, may account for much of the association between greater overall screen use and executive-function diddiculty, whereas device-specific associations require further replication.

One potential explanation for the activity-specific association is that viewed content can provide externally paced audiovisual stimulation and sequencing, which may place fewer demands on children to actively direct and sustain their own attention. An experimental study showed that even brief exposure to fast-paced television can temporarily impair executive-function performance in preschool-aged children.^40^ Digital games, in contrast, are interactive and require children to make behavioral responses to on-screen content. However, our cross-sectional design cannot establish whether greater viewing precedes executive-function diddiculty, whether children with weaker executive function are given greater screen access, or whether both reflect shared child and family characteristics.

### Emotional self-regulation and social-emotional development.

This is one of the few studies to examine emotional self-regulation specifically,^41^ as distinct from broader social-emotional functioning.^5,42^ Total screen time was not associated with emotional-regulation diddiculty; however, decomposition by device revealed that mobile-device use was associated with poorer emotional self-regulation (β = 0.13 SD; r = 0.21) whereas television use was not, consistent with prior studies.^22,43,44^ A parallel pattern was observed for broader social-emotional functioning, with greater mobile-device use associated with higher SDQ total diddiculty scores (β = 0.09 SD; r = 0.15), while television use was not.

Notably, the ERC Lability/Negativity subscale, which captures mood swings, angry reactivity, and inflexible, poorly modulated negative affect,^24^ was associated with mobile-device use, whereas the ERC Emotion Regulation subscale was not. Although not specific to activity type, a prior study similarly reported an association of greater total screen time with higher ERC Lability/Negativity subscale scores, but not with the ERC Emotion Regulation subscale scores.^35^ Our findings suggest that mobile-device use may be more closely related to children’s tendency toward intense or labile negative emotional responses than to their broader capacity for emotion regulation. One possible explanation is that the portability and immediate availability of mobile devices make them particularly easy for caregivers to use during moments of child distress. Consistent with this possibility, Radesky et al.^18^ reported longitudinal bidirectional associations between the use of mobile devices for calming and subsequent emotional reactivity among children aged 3–5 years. Similarly, Coyne et al.^46^ found that greater use of media for emotion regulation was associated with poorer emotional knowledge and empathy and greater emotional reactivity. Repeated reliance on mobile media during distress could reduce opportunities for children to practice internally driven regulation strategies and for caregivers to scaffold emotion regulation through interaction. Conversely, children with greater emotional reactivity may be more likely to receive mobile devices as a calming strategy.^15^ Thus, the observed association may reflect a bidirectional process; however, the cross-sectional design of the present study precludes establishing temporal ordering.

### Child screen media use and family media context.

The findings described above point to specific and modifiable features of screen media use: video viewing in relation to executive functioning and mobile-device use in relation to emotional self-regulation. The findings regarding the family media context provide additional insight into how these patterns of screen use may occur in everyday family settings. As expected, video viewing was moderately correlated with more frequent background television (ρ = 0.48), partly reflecting overlap between these constructs, as background television offers opportunities for children to view television content.^49,50^ Similarly, the moderate correlation between mobile-device use and parental use of screens to calm the child (ρ = 0.47) is consistent with more frequent use of mobile devices in the context of managing child distress.^18,46^ These contextual correlations identify specific family media practices, including background television and use of screens for calming, that warrant examination in a prospective design. Future studies should evaluate whether these family media practices shape children’s screen use patterns and whether those patterns in turn predict the development of executive functioning and emotional self-regulation.

### Limitations.

Several limitations should be considered. First, this cross-sectional study precludes establishing the temporal ordering of screen media use and developmental outcomes. Second, children’s screen media and developmental outcomes were reported by mothers, which may be subject to recall and social desirability bias. Third, although targeted community outreach enabled us to enroll approximately two thirds of participants from lower-to-moderate-resource neighborhoods, participating mothers were predominantly college-educated (79%), which limits generalizability to other populations. In addition, because BEES used convenience sampling, the sample is not representative of the target population in the geographic recruitment area. Fourth, ERC administration began partway through enrollment, resulting in missing ERC data for 52 initial participants. Because this missingness was determined by date of enrollment rather than by any participant characteristic, it is unlikely to have substantially biased the observed association between screen media use and ERC. Finally, residual confounding by unmeasured family and child characteristics cannot be ruled out.

## Conclusions

In a sample of U.S. preschool-aged children, associations between screen media use and developmental outcomes varied by activity and device type. Video viewing was associated with greater executive-function diddiculty, whereas mobile-device use was associated with greater emotional-regulation diddiculty. Screen use occurred within distinct family media contexts: video viewing was correlated with more frequent background television, whereas mobile-device use was correlated with more frequent use of screens to calm the child. Findings highlight the importance of considering activity and device type when examining the impact of screen media use on child development.

## Supplementary Material

Supplementary Files

This is a list of supplementary files associated with this preprint. Click to download.

- SupplementaryData9.25.2026.docx

## Figures and Tables

**Figure 1:**
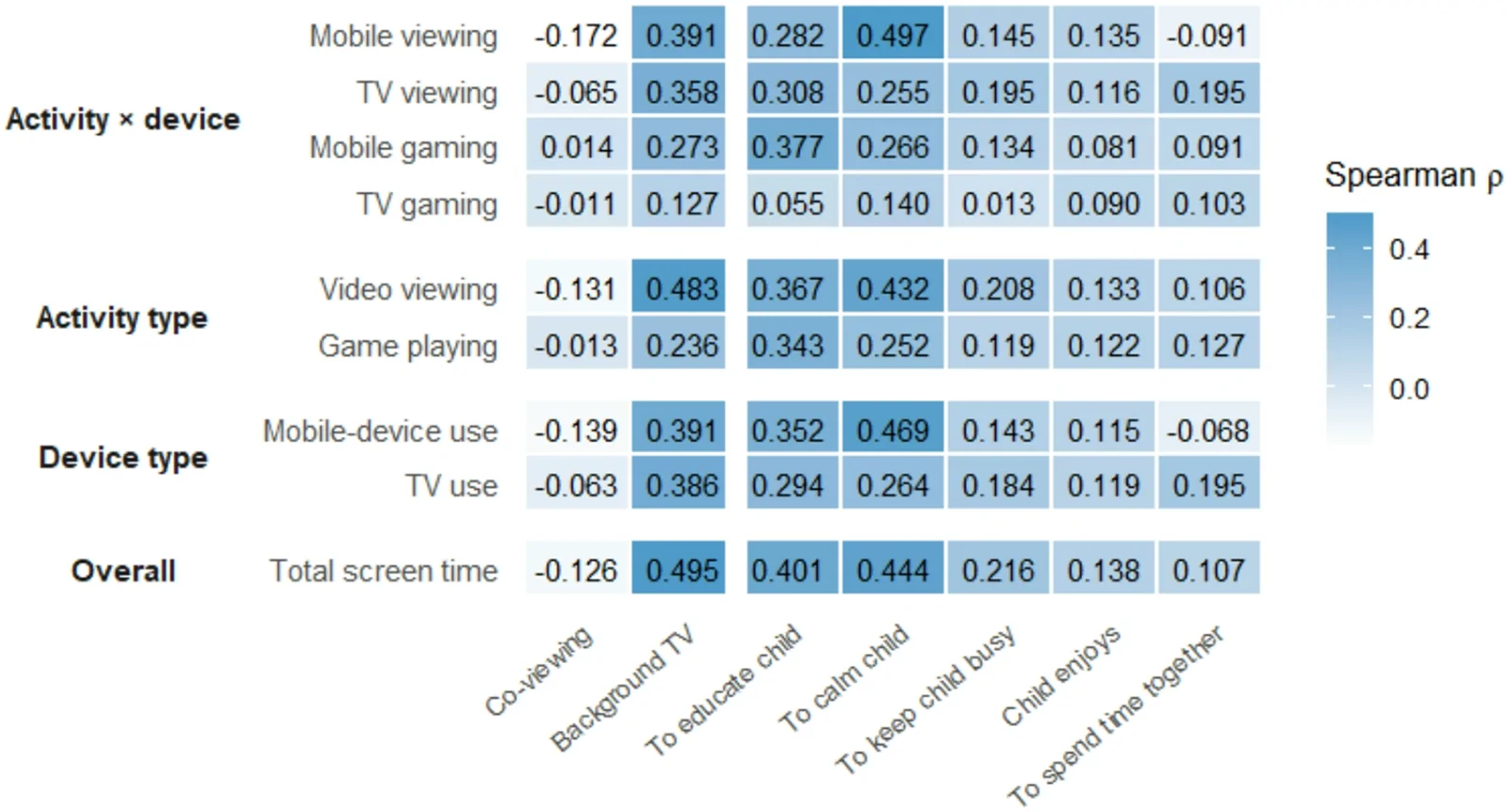
Correlations between screen media use and family media context variables

**Figure 2:**
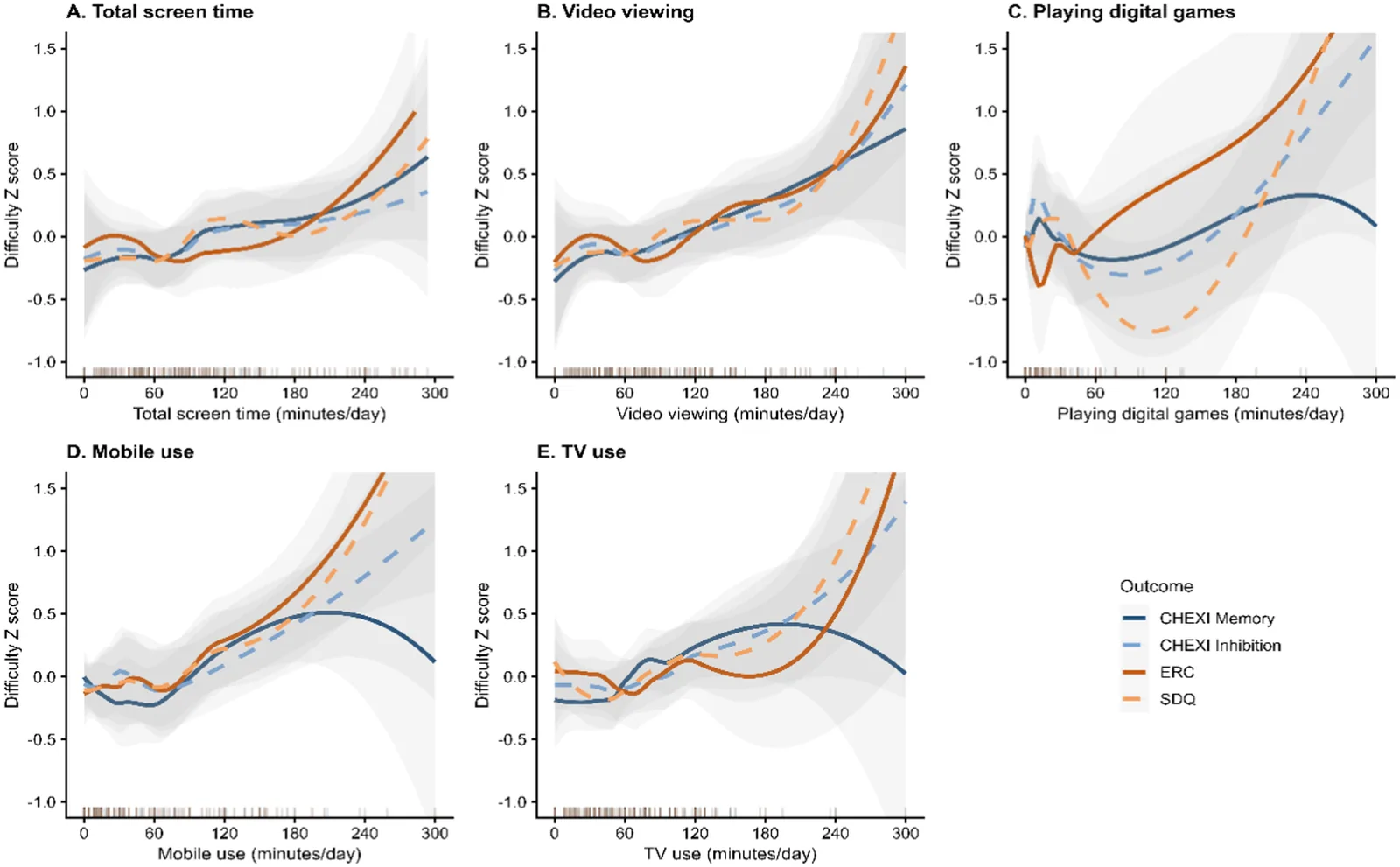
Locally estimated scatterplot smoothing (LOESS) curves for screen media exposures and developmental outcomes LOESS curves are presented up to 300 minutes/day of screen media use. The game time curve (panel C) was unstable with large 95% confidence intervals, as over a half of the participants had zero minutes for digital game play. Device-specific curves (panels D and E) were also unstable at the extreme tails as only three participants had ≥240 minutes/day.

**Table 1:** Participant characteristics

Variable	N (%)
Total	238
**Age**	
3 years old	185 (77.7)
4 years old	53 (22.3)
**Sex**	
Male	118 (49.6)
Female	120 (50.4)
**Race and ethnicity**	
Hispanic	70 (29.4)
Non-Hispanic Asian/Pacific Islander	9 (3.8)
Non-Hispanic Black	30 (12.6)
Non-Hispanic White	104 (43.7)
Non-Hispanic multiracial	25 (10.5)
**Maternal education**	
<4-year college degree	48 (20.2)
≥4-year college degree	190 (79.8)
**Maternal employment**	
Employed	187 (78.6)
Not employed	51 (21.4)
**Maternal marital status**	
Married	177 (74.4)
Not married	61 (25.6)
**Childcare attendance**	
Yes	190 (79.8)
No	48 (20.2)
**Child Opportunity Index**	
Very low	29 (12.2)
Low	63 (26.5)
Moderate	58 (24.4)
High	34 (14.3)
Very high	54 (22.7)
Screen exposure, minutes/day	Mean ± SD
Video viewing on mobile devices	29.71 ± 39.16
Video viewing on TV	60.27 ± 39.64
Playing digital games on mobile devices	13.12 ± 24.93
Playing digital games on TV	5.59 ± 19.90
Overall screen time	108.69 ± 92.01

**Table 2:** Comparison of developmental outcomes between high and low total screen time categories

Outcome	All	Total screen time ≤ 1 hour/day (n = 90)	High screen time > 1 hour/day (n = 148)	P value
	Mean ± SD	Mean ± SD	Mean ± SD	
CHEXI instrument				
CHEXI working memory difficulty score	2.05 ± 0.66	1.95 ± 0.55	2.12 ± 0.71	0.04
CHEXI inhibition difficulty score	2.65 ± 0.79	2.57 ± 0.73	2.70 ± 0.82	0.23
CHEXI executive-function difficulty score	2.29 ± 0.64	2.20 ± 0.56	2.35 ± 0.69	0.06
ERC instrument				
ERC emotion dysregulation score	1.48 ± 0.37	1.53 ± 0.41	1.45 ± 0.35	0.13
ERC liability/negativity score	1.77 ± 0.43	1.73 ± 0.40	1.79 ± 0.45	0.30
ERC emotion- regulation difficulty score*	1.67 ± 0.35	1.66 ± 0.33	1.68 ± 0.36	0.76
SDQ instrument				
SDQ externalizing score	5.47 ± 3.82	4.88 ± 3.46	5.82 ± 4.00	0.06
SDQ internalizing score	3.71 ± 1.92	3.58 ± 1.84	3.80 ± 1.97	0.39
SDQ total difficulty score	9.18 ± 5.04	8.46 ± 4.44	9.62 ± 5.34	0.08
SDQ peer problem score	2.52 ± 1.14	2.38 ± 1.06	2.61 ± 1.18	0.13

* ERC sample: total n = 186; n = 74 for ≤ 1 hour; n = 112 for > 1 hour. P values are from two-sample t tests. Statistical significance was evaluated using a Bonferroni-adjusted threshold of *p* < 0.025.

CHEXI, Childhood Executive Functioning Inventory; ERC, Emotion Regulation Checklist; SD, standard deviation; SDQ, Strengths and Difficulties Questionnaire.

**Table 3:** Multivariable models for associations of total screen time with developmental outcomes

Variable	CHEXI executive-function difficulty score		ERC emotional-regulation difficulty score		SDQ total difficulty score	
	B (95% CI)	P value	B (95% CI)	P value	B (95% CI)	P value
Age per year	0.11 (−0.11, 0.33)	0.34	−0.07 (−0.23, 0.09)	0.38	0.55 (−1.17, 2.27)	0.53
Sex: male vs. female	0.18 (0.01, 0.34)	0.03	0.14 (0.04, 0.24)	**0.006**	1.33 (0.05, 2.61)	0.04
Maternal education: < vs. ≥4-year college degree	0.08 (−0.15, 0.30)	0.50	0.12 (−0.02, 0.25)	0.10	0.71 (−1.02, 2.44)	0.42
COI: low vs. high	0.00 (−0.21, 0.20)	0.98	−0.02 (−0.15, 0.10)	0.71	0.71 (−0.88, 2.30)	0.38
COI: moderate vs. high	0.05 (−0.16, 0.27)	0.63	−0.08 (−0.22, 0.05)	0.22	0.39 (−1.30, 2.07)	0.65
Additional 30 min/day screen time	0.03 (0.01, 0.06)	**0.02**	0.01 (0.00, 0.03)	0.12	0.27 (0.04, 0.49)	**0.02**

Statistical significance was evaluated using a Bonferroni-adjusted threshold of *p* < 0.025; significant associations are shown in bold.

CHEXI, Childhood Executive Functioning Inventory; CI, confidence interval; COI, Child Opportunity Index; ERC, Emotion Regulation Checklist; SD, standard deviation; SDQ, Strengths and Difficulties Questionnaire.

**Table 4:** Multivariable models for associations of video viewing and digital game playing with developmental outcomes

Variable	CHEXI executive-function difficulty score		ERC emotional-regulation difficulty score		SDQ total difficulty score	
	B (95% CI)	P value	B (95% CI)	P value	B (95% CI)	P value
Age per year	0.11 (−0.11, 0.33)	0.31	−0.07 (−0.22, 0.09)	0.40	0.51 (−1.21, 2.23)	0.56
Sex: male vs. female	0.18 (0.02, 0.34)	0.03	0.14 (0.04, 0.24)	**0.006**	1.33 (0.05, 2.61)	0.04
Maternal education: < vs. ≥4-year college degree	0.08 (−0.14, 0.30)	0.48	0.11 (−0.03, 0.25)	0.12	0.69 (−1.04, 2.42)	0.43
COI: low vs. high	−0.01 (−0.21, 0.19)	0.93	−0.02 (−0.15, 0.10)	0.70	0.75 (−0.85, 2.34)	0.36
COI: moderate vs. high	0.05 (−0.16, 0.27)	0.63	−0.09 (−0.22, 0.05)	0.20	0.39 (−1.29, 2.08)	0.65
Additional 30 min/day video viewing	0.06 (0.01, 0.11)	**0.01**	0.00 (−0.03, 0.03)	0.85	0.13 (−0.24, 0.50)	0.50
Additional 30 min/day digital game playing	−0.01 (−0.08, 0.06)	0.78	0.05 (0.00, 0.10)	0.05	0.52 (−0.05, 1.08)	0.07

Statistical significance was evaluated using a Bonferroni-adjusted threshold of *p* < 0.025; significant associations are shown in bold.

CHEXI, Childhood Executive Functioning Inventory; CI, confidence interval; COI, Child Opportunity Index; ERC, Emotion Regulation Checklist; SD, standard deviation; SDQ, Strengths and Difficulties Questionnaire.

**Table 5:** Multivariable models for associations of mobile-device and TV use with developmental outcomes

Variable	CHEXI executive-function difficulty score		ERC emotional-regulation difficulty score		SDQ total difficulty score	
	B (95% CI)	P value	B (95% CI)	P value	B (95% CI)	P value
Age per year	0.11 (−0.11, 0.33)	0.34	−0.06 (−0.21, 0.10)	0.48	0.60 (−1.12, 2.31)	0.49
Sex: male vs. female	0.18 (0.02, 0.34)	0.03	0.14 (0.04, 0.24)	**0.005**	1.36 (0.08, 2.64)	0.04
Maternal education: < vs. ≥4-year college degree	0.07 (−0.15, 0.30)	0.52	0.11 (−0.02, 0.25)	0.10	0.64 (−1.09, 2.37)	0.47
COI: low vs. high	0.00 (−0.21, 0.20)	0.97	−0.03 (−0.16, 0.10)	0.64	0.67 (−0.92, 2.26)	0.41
COI: moderate vs. high	0.05 (−0.16, 0.27)	0.62	−0.08 (−0.21, 0.05)	0.22	0.41 (−1.27, 2.09)	0.63
Additional 30 min/day mobile-device use	0.04 (−0.01, 0.09)	0.12	0.05 (0.01, 0.08)	**0.004**	0.47 (0.07, 0.86)	**0.02**
Additional 30 min/day TV use	0.03 (−0.03, 0.09)	0.34	−0.02 (−0.06, 0.01)	0.23	0.03 (−0.42, 0.48)	0.89

Statistical significance was evaluated using a Bonferroni-adjusted threshold of p < 0.025; significant associations are shown in bold.

CHEXI, Childhood Executive Functioning Inventory; CI, confidence interval; COI, Child Opportunity Index; ERC, Emotion Regulation Checklist; SD, standard deviation; SDQ, Strengths and Difficulties Questionnaire.

## Data Availability

Data may be available upon request to the corresponding author: Soyang Kwon, soyang.kwon@northwestern.edu.

## List of abbreviations

BEES: Behavioral and Emotional Development in Early Childhood Study
CAMPAS: Child and Mother Physical Activity Study
CHEXI: Childhood Executive Functioning Inventory
CI: Confidence interval
COI: Child Opportunity Index
ERC: Emotion Regulation Checklist
LOESS: locally estimated scatterplot smoothing
MAQ: Media Assessment Questionnaire
SD: standard deviation
SDQ: Strengths and Difficulties Questionnaire
U.S.: United States
